# Episodic Breathlessness with and without Background Dyspnea in Advanced Cancer Patients Admitted to an Acute Supportive Care Unit

**DOI:** 10.3390/cancers12082102

**Published:** 2020-07-29

**Authors:** Sebastiano Mercadante, Claudio Adile, Patrizia Ferrera, Giuseppe Bonanno, Vincenzo Restivo, Alessandra Casuccio

**Affiliations:** 1 Main Regional Center for Pain Relief and Palliative/Supportive Care, La Maddalena Cancer Center, 90145 Palermo, Italy; claudio.adile@hotmail.it (C.A.); patriziaferrera@inwind.it (P.F.); giuseppebonanno91@gmail.com (G.B.); 2 Department of Health Promotion Sciences, Maternal and Infant Care, Internal Medicine and Medical Specialties (PROMISE), University of Palermo, 90130 Palermo, Italy; vincenzo.restivo@unipa.it (V.R.); alessandra.casuccio@unipa.it (A.C.)

**Keywords:** dyspnea, episodic breathlessness, opioids, palliative care, advanced cancer

## Abstract

Aim: To characterize episodic breathlessness (EB) in patients with advanced cancer, and to determine factors influencing its clinical appearance. Methods: A consecutive sample of advanced cancer patients admitted to an acute palliative care unit was surveyed. Continuous dyspnea and EB were measured by a numerical scale. The use of drugs used for continuous dyspnea and EB was recorded. Patients were asked about the characteristics of EB (frequency, intensity, duration and triggers). The Multidimensional dyspnea profile (MDP), the Brief dyspnea inventory (BDI), the Athens sleep scale (AIS) and the Hospital Anxiety and Depression Scale (HADS) were also administered. Results: From 439 advanced cancer patients surveyed, 34 and 27 patients had EB, without and with background dyspnea, respectively. The mean intensity and the number of episodes were higher in patients with background dyspnea (*p* < 0.0005 and *p* = 0.05, respectively). No differences in duration were observed. Most episodes lasted <10 min. A recognizable cause triggering EB was often found. The presence of both background dyspnea and EB was associated with higher values of MDP and BDI. EB was independently associated with frequency and intensity of background dyspnea (OR = 20.9, 95% CI (Confidence interval) 9.1–48.0; *p* < 0.0005 and OR = 1.97, 95% CI 1.09–3.58; *p* = 0.025, respectively) and a lower Karnofsky level (OR = 0.96, 95%CI 0.92–0.98, *p* = 0.05). Discussion: EB may occur in patients with and without continuous dyspnea, and is often induced by physical and psychological factors. EB intensity is higher in patients with continuous dyspnea. The duration was often so short that the use of drugs, as needed, may be too late, unless administered pre-emptively when the trigger was predictable.

## 1. Introduction

Dyspnea is a very distressing symptom often reported in advanced cancer patients, particularly in the last months of life [1]. Dyspnea is associated with relevant suffering, both in cancer patients and caregivers. Although patients with lung cancer are commonly considered at higher risk to develop dyspnea, this symptom may largely occur in other primary tumors [2]. Dyspnea has been defined as an unpleasant sensation in or experience of breathing, characterized by qualitatively distinct sensations presenting with variable intensity [3].

Dyspnea is often a continuous phenomenon present for most hours of the day. However, this symptom may also occur abruptly due to known and unknown triggering factors, or may aggravate or overlap with the background condition, with peaks of intensity clearly distinguishable from the background level, resembling the phenomenon of breakthrough cancer pain [4]. This phenomenon is commonly named episodic breathlessness (EB) [5]. On the other hand, EB may develop without significant background dyspnea, mainly due to the activation of triggering factors.

In the last few years, EB has been differently characterized, and more information is now available. As EB is a complex entity demanding complex management, it is of paramount importance to gather data about this phenomenon in order to optimize the treatment and to provide better and individualized therapeutic options [5,6,7,8,9].

In previous papers, the prevalence of dyspnea and EB were surveyed in advanced cancer patients recruited in different settings. However, the selection of patients was based on the presence of background dyspnea. Moreover, only physical factors were taken into consideration, excluding possible psychological factors as triggering agents for EB [8,9]. The aim of this study was to better characterize EB, in the presence or not of background dyspnea. The secondary outcome was to explore factors that may influence its clinical appearance.

## 2. Patients and Methods (Ethical Code: n.1/2014)

A consecutive sample of advanced cancer patients admitted to an acute palliative care unit was surveyed for a period of 18 months. This unit is characterized by the admission of patients who often receive anticancer therapies and require pain and symptom management. Thus, this cohort of cancer patients are admitted in a relatively early stage of disease, and in some cases are admitted before staring a treatment. The activities of this unit have been described elsewhere [10].

Inclusion criteria were age ≥18 years, a diagnosis of cancer, and the ability to provide information about continuous dyspnea and EB. Exclusion criteria were the incapacity to complete the questionnaires for any reason, relevant cognitive failure (Memorial Delirium Assessment Scale (MDAS) ≥13, on a scale 0–30), any refusal, and a Karnofsky level of ≤20 (on a scale 0–100). Patients’ informed consent and institutional approval from the University of Palermo were obtained.

### 2.1. Data Collection

At-admission age, gender, primary tumor, Karnofsky level, stage of disease, concomitant comorbidities and oncologic treatments were recorded. Patients meeting inclusion and exclusion criteria were asked about the presence of any form of dyspnea. If the answer was yes, assessment was continued. The palliative prognostic score (PaP) was calculated. The PAP uses the Karnofsky Performance Score (KPS) and five other criteria to generate a numerical score from 0 to 17.5 (higher scores predicting shorter survival) [11]. Continuous dyspnea, defined as a sensation of breathlessness for most hours of the day, was measured by the Edmonton Assessment Symptom scale (ESAS) over the previous 24 h. The ESAS consists of 10 items, including dyspnea, which are rated 0 to 10, evaluating the intensity of physical and psychological symptoms and the sense of poor well-being [12]. The use of drugs for continuous dyspnea was recorded.

The definition of EB was made according to an agreement among experts, and included: the intermittent, time-limited character of episodes; the subjective experience and qualitative aspects of breathing discomfort that differentiate episodic from continuous breathlessness in general; and predictability (triggered by identifiable factors) [7]. Patients were asked about the number of episodes/day (clearly distinguished from baseline dyspnea, if any), intensity, and duration of untreated episodes. Other information included the eventual triggers (physical activity, going upstairs, walking, movement in the bed, supine position), if dyspnea hindered every day and favorite activities, or these activities did not solve it or prevent it, if anxiety induced EB and vice versa (if EB induced anxiety), and daily prevalence (diurnal or nocturnal) of EB. Information on drugs used for continuous dyspnea and EB, and their efficacy (effective, moderately effective, ineffective), were collected.

### 2.2. Measurements

The Multidimensional dyspnea profile (MDP), the Brief dyspnea inventory (BDI), the Athens sleep scale (AIS) and the Hospital Anxiety and Depression Scale (HADS) were administered. The MDP is a tool developed to separately measure the immediate unpleasantness or discomfort of breathing, the presence and intensity of five sensory quality items, and the intensity of five emotional responses to breathlessness (for each item, the score is 0–10) [13,14]. The BDI is a valid and reliable measure for evaluating dyspnea, and to inform about the relative severity and interference of dyspnea in daily life activities (7 items, score 0–10) [15]. The AIS is a self-assessment psychometric instrument designed for quantifying sleep difficulty. It consists of eight items: the first five items pertain to sleep induction, awakenings during the night, final awakening, total sleep duration and sleep quality; while the last three refer to well-being, functioning capacity and sleepiness during the day [16]. Each item is scored from 0 to 3. The HADS is a self-assessment tool and consists of 14 items, with two separate subscales for anxiety and depression. The total score ranges from 0 to 42, with a higher score indicating severe depression and anxiety [17].

### 2.3. Statistical Analysis

Summary statistics for dyspnea, continuous and categorical variables, and the associated frequency distributions have been provided. Primary tumors have been categorized into 12 categories. The variable age has been categorized, to improve its interpretability and model implementation, into four categories, ranging from an age less than 66 years to an age more than 85 years. Categorical variables were described as absolute (n) and relative (%) frequencies; mean and standard deviation (SD) summarized the continuous variables. χ2 explorative cross-tabulation tests of association among variables, assuming α = 0.05, were carried out.

Differences between the clinical characteristics of patients with dyspnea and EB dyspnea were assessed by the chi-square test or Fisher exact test, as needed for categorical variables, and by the independent Student t test for continuous parameters if the data were normally distributed. The differences between time intervals were determined via paired *t*-test.

All variables that were found to be significantly associated (*p* ≤ 0.05) in the univariate analysis were included in a multivariate regression model. Data were analyzed by IBM SPSS Software 22 version (IBM Corp., Armonk, NY, USA). All *p*-values were two-sided and *p* < 0.05 was considered statistically significant.

## 3. Results

A total of 439 advanced cancer patients admitted to the acute pain relief and supportive/palliative care unit were surveyed in the study period. Of the initial patients screened, 40 patients reported background dyspnea. One patient was then excluded for inability to provide further data. The mean Karnofsky status recorded at admission was 51.3 (SD = 9.5). The characteristics of patients are shown in Table 1. Lung cancer, bronchopulmonary diseases and palliative care treatment were statistically associated with dyspnea. The mean values of ESAS items (SD) are reported in Table 2.

**Figure 1:**
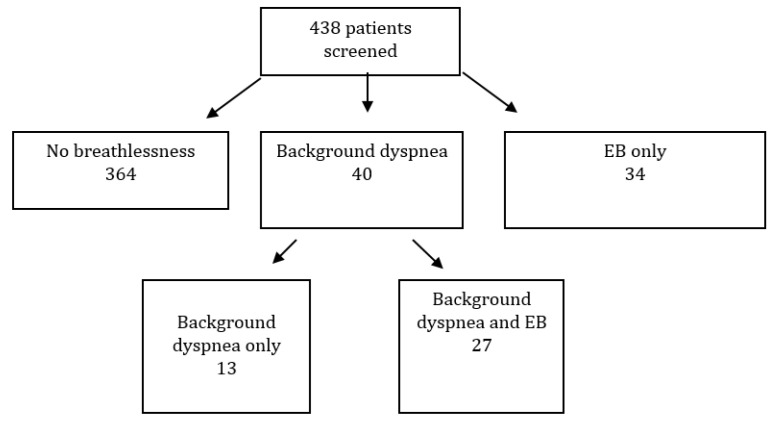
Flow chart of patients’ distribution. EB = episodic breathlessness.

A total of 74 patients reported background dyspnea, EB, or both. In total, 38 (8.7%), 26 (5.9%) and 4 (0.9%) patients belonged to PaP risk groups A, B and C, respectively (data were missing for some patients). Of the 40 patients with background dyspnea, 27 (67.5%) had EB. A total of 34 patients had EB without reporting background dyspnea (Figure 1).

### 3.1. Background Dyspnea

The prevalence of background dyspnea was 9.1% (40/839). In such patients, the mean intensity of dyspnea was 5.9 (SD 2.1). A total of 22 patients (55%) were receiving drugs for background dyspnea, in the rank order corticosteroids (n.10, 25%), bronchodilatators (n.5, 12.5%), oxygen (n.3, 7.5%), diuretics (n.2, 5%) and opioids (n.1, 2.5%). Associated comorbidities (*n* = 34, 85%) were in the rank order cardiovascular disease (70.6%), chronic obstructive pulmonary disease (32.3%), renal failure (5.9%), and others (52.9%).

Background dyspnea was significantly associated with cancer diagnosis (>in lung cancer, *p* < 0.0005, Fisher’s exact test, <in gastrointestinal and pancreatic cancer, *p* = 0.003 and *p* = 0.014, respectively; Fisher’s exact test), comorbidities (bronchopulmonary, *p* = 0.010, Fisher’s exact test) and type of treatment (palliative care, *p* < 0.0005, Fisher’s exact test) (Table 1). According to the multinomial logistic regression analysis, the risk of background dyspnea increased with the diagnosis of lung cancer (OR 1.14, 95%CI 1.02–1.27, *p* = 0.019).

### 3.2. Episodic Breathlessness

In total, 34 patients and 27 patients had EB without and with background dyspnea, respectively. The mean intensities of EB were 5.8 (SD 2.2) and 7.7 (SD 1.8) in patients without and with background dyspnea, respectively. The difference was statistically significant (*p* < 0.0005, *t*-test). The mean number of episodes/day was 3.4 (SD 2.3, range 1–15). The number of episodes was significantly higher in patients with background dyspnea (*p* = 0.05).

In the 27 patients with background dyspnea, the mean durations of EB were <5 min, 5–10, 11–20 and >20 min in 14, 6, 5 and 1 patients, respectively (data missing for one patient). In the 34 patients without background dyspnea, the mean durations of EB were <5 min, 5–10, 11–20 and >20 min, in 17, 8, 2 and 5 patients, respectively (missing data for 2 patients). The difference was not significant (*p* = 0.267, chi square test).

In all patients, a recognizable cause triggering EB was found, as follows: going up the stairs (*n* = 35), walking (*n* = 44), movement in the bed (*n* = 18), supine position (*n* = 16) and psychological factors (anxiety, panic) (*n* = 46). In 43 patients, EB upon movement limited or prevented daily activities. In 27 patients, anxiety induced EB, while in 32 patients EB induced anxiety. Finally, in 19 patients the influence was reciprocal. EB prevalence was diurnal, nocturnal or both, in 31, 11 and 18 patients, respectively. In total, 17 patients were receiving drugs for EB, including bronchodilatators (*n* = 7), corticosteroids (*n* = 4), oxygen (*n* = 3), diuretics (*n* = 1) and others (*n* = 2). The efficacy was high, mild and poor in 1, 9 and 3 patients, respectively.

The mean MDP was 6.1 (SD 2.4) for muscle effort; 6.7 (SD 2.3) for unpleasantness; 24.1 (SD 11) for quality-intensity, and 20.1 (SD 14.2) for emotional responses to dyspnea. The mean BDI was 38.2 (SD 15.6). The mean ASS was 9.1 (SD 4.7). The mean HADS-A was 8.9 (SD 4.1). The mean HADS-D was 9.6 (SD 3.9), while HADS total was 18.5 (SD 6.6).

The differences in MDP items, ASS, HADS-A, HADS-D and total HADS, in patients with background dyspnea, background dyspnea and EB, and EB alone, are reported in Table 3. Patients with background dyspnea and EB had the highest values of MDP items and BDI.

In the univariate analysis, EB was associated with the frequency (*p* < 0.0005) and intensity of background dyspnea (*p* = 0.007), a diagnosis of lung cancer (*p* < 0.0005), a lower Karnofsky level (*p* = 0.020), bronchopulmonary comorbidities (*p* = 0.045), palliative care treatment (*p* < 0.0005), MDP effort (*p* = 0.007) and MDP unpleasantness (*p* = 0.006). In the multivariate analysis, EB was independently associated with frequency and intensity of background dyspnea (OR = 20.9, 95%CI 9.1–48.0; *p* < 0.0005 and OR = 1.97, 95%CI 1.09–3.58; *p* = 0.025, respectively) and a lower Karnofsky level (OR = 0.96, 95%CI 0.92–0.98, *p* = 0.05).

## 4. Discussion

The principal findings of this study were that patients may develop EB with or without background dyspnea, and that patients having background dyspnea or a lower Karnofsky status developed more, and more severe, EB episodes. EB was often induced by physical and psychological factors. Of interest, the prevalence of background dyspnea was low in this cohort of patients.

### 4.1. Background Dyspnea

A lower prevalence of background dyspnea (less than 10%) was found in comparison with the previous data reported in the literature, which possibly depended on different stages of disease and settings [1,2,18,19]. In home palliative care patients, presumably having a short life expectancy, the prevalence of dyspnea was about 35%, and the Karnofsky level was noticeably lower [19]. In another study performed in inpatients and outpatients, constant dyspnea accounted for 39% of subjects who had a median Karnofsky of 80. Of these patients, 20% also experienced episodes of EB [20]. This difference could be due to the different settings and methodologies. This data suggests that patients included in this analysis were admitted early in the course of their disease, according to the characteristics of this unit previously described [10], as confirmed by the relatively higher level of Karnofsky status recorded at admission. The second point regarding chronic dyspnea was that opioids were given to only one patient. Opioids are often given in more advanced stages of disease, although the best evidence supports the use of opioids for breathlessness in opioid-naïve patients [18].

As expected, some factors, such as lung cancer, chronic respiratory disease and no anticancer treatment, were found to be associated with background dyspnea, although only lung cancer was independently associated with background dyspnea.

### 4.2. Episodic Breathlessness

Of the 40 patients with background dyspnea, 27 (67%) experienced EB episodes, while 37 patients experienced EB alone. In a previous study, continuous dyspnea accounted for 27 (39%) patients, and among them 14 (20%) patients also presented episodes of EB. EB alone occurred in 43 (61%) of patients [20]. A similar categorization has been proposed, adding further elements of complexity when evaluating EB [7]. Of interest, more episodes were reported in patients with background dyspnea, resembling patients with breakthrough pain, wherein pain episodes are more likely to occur in patients with higher levels of background pain [4]. This observation also supports the optimization of background dyspnea with an appropriate treatment, possibly anticipating the use of opioids. This aspect deserves future studies.

### 4.3. Triggers for Episodic Breathlessness

In different chronic lung diseases, EB on movement limited or prevented the daily activity. EB triggered by exertion, non-triggered EB and continuous dyspnea have been found to be the most frequent and important categories, with a high impact on daily living. Exertional EB occurred in nearly all patients [5,6]. Similar to other studies [7,20], EB prevalence was prevalently distributed during the day, although episodes also occurred at night, when physical activity is commonly minimal. Many episodes were triggered by psychological factors. This aspect has not been explored in previous studies [8,9], and has not been specifically assessed in other studies. Of interest, anxiety may circularly be produced by the EB episode itself. Similar to EB induced by physical activity, the preventive use of anxiolytics around the clock, or non-pharmacologic techniques, may be helpful in preventing episodes triggered by fear or panic. These aspects deserve further exploration.

### 4.4. Episodic Breathlessness Intensity

EB intensity in patients with background dyspnea was shown to be higher than in patients without dyspnea. This finding has never been reported in previous studies, in which a mean intensity of 6.5 was reported, regardless of the background dyspnea intensity [20,21]. This can be explained by the level of tolerance expressed by these patients, who are likely to require higher levels of EB intensity to feel a clear difference from their baseline dyspnea.

### 4.5. Episodic Breathlessness Duration 

From a clinical perspective, EB duration has an obviously relevant role. Most events lasted less than 5 min, and 78% of patients reported an EB duration of ≤10 min, with no differences between patients with or without continuous dyspnea. This data is consistent with previous experiences in patients with different diseases, in which an EB duration of less than 10 min was found in 75–88% of cases, the majority being less than 5 min [20,21,22], and with only 9% of episodes lasting more than 20 min. The short duration of EB refers us back the therapeutic options, commonly based on the administration of drugs like opioids, with a short onset. It is hard to argue that there would be a treatment effective in such a short time. In a randomized, placebo-controlled, double-blind, double-dummy study, small doses of nebulized hydromorphone, systemic hydromorphone and nebulized saline in opioid-tolerant patients did not differ in their ability to improve EB 10 min after administration, although a clinical improvement of at least one point was achieved with nebulized hydromorphone. Breathlessness scores decreased linearly from 10 to 60 min post-treatment completion [23]. In a randomized cross-over study performed in a real-life clinical setting, focused on exertional EB, the mean times to onset for fentanyl buccal tablet and oral morphine were 12.7 and 23.6 min, respectively [24]. Considering that about 80% of episodes last ≤10 min, it is likely that the effect is produced by the natural course of EB, with a spontaneous disappearance, particularly when EB is induced by an effort, possibly stopped after EB occurred [25].

It seems evident that a better option would be the pre-emptive use of drugs, like short-onset opioids, for predictable events, rather than a treatment once the episode occurred, as most episodes will vanish before any treatment can provide a clinical effect. This is confirmed by the finding that most episodes were triggered by predictable physical efforts. A series of studies demonstrated that walking-induced EB may be reduced by the prophylactic use of transmucosal or subcutaneous fentanyl, which demonstrated a dose–response relationship in improving both dyspnea and walking distance [26,27,28,29]. Of interest, the high-dose groups (35–45% of the daily background equivalent opioid dose) were more likely to report at least somewhat better improvement, without producing significant adverse events, confirming the protective role of the level of opioid tolerance reported for breakthrough pain [4]. Considering that oral opioids take about 30 min, and fast-acting drugs about 10 min, to provide a clinical effect, these options could be inappropriate for these short-lasting episodes, unless used pre-emptively, before a predictable effort. Nonpharmacological strategies (e.g., panic control techniques, distraction or hand-held fan) could be accessible at any time, and might be helpful. Patients may use a number of different strategies to cope with EB, including a reduction of physical exertion, a change of position, laying down flat or standing up and raising the hands, environmental strategies, concentrating on the breathing or distracting from any thoughts of breathlessness, breathing techniques, air and oxygen, or cognitive and psychological strategies [30]. However, these strategies need more specification and an appropriate clinical evaluation for the management of EB.

### 4.6. Factors Associated with Continuous Dyspnea and Episodic Breathlessness

No differences were found for patients with sleep disturbances or psychological factors, assessed by ASS and HADS, among the categories of dyspnea with EP, without EB and EB alone. Indeed, patients with both background dyspnea and EB had the highest values of MDP items and BDI. This finding suggests that the unpleasantness or discomfort of breathing, the presence and intensity of sensory quality items, emotional responses to breathlessness, and interference in daily life activities are more likely to develop in this category of patients. Thus, this group of patients develops the greatest global burden, compared to patients with EB or background dyspnea alone. In a previous study performed on patients with cardiopulmonary diseases, severity and unpleasantness were found to be associated with worse perceived quality of life, age, gender, diagnosis, morning/evening assessment, breathlessness score, morphine treatment and the presence of different sensory qualities of breathlessness [14].

The principal limitation of this study involves the specific characteristics of the acute palliative care unit, where only cancer patients are admitted, relatively early for palliative care, and for different reasons. This explains the low number of patients with dyspnea who were recruited, in comparison with the prevalence of dyspnea observed in other studies. The explorative design limits the generalization of our results. No sample size was calculated beforehand and the categories tested were built post hoc. Therefore, the present results need to be interpreted with caution.

## 5. Conclusions

EB may occur in patients with and without continuous dyspnea, and it is often induced by physical and psychological factors. EB intensity is greater in patients with continuous dyspnea. The duration is relatively small, limiting the use of drugs as needed, unless they are administered pre-emptively when the trigger is predictable. These findings encourage the improvement of the level of background dyspnea, or the finding of an alternative measure for EB. Patient-relevant therapeutic targets for the future evaluation of adequate pharmacological and nonpharmacological management options should be determined in future studies.

## Figures and Tables

**Table 1:** Characteristics of patients screened for the study.

			No Dyspnea N° 398	Dyspnea N° 40	
Age		≤65 years	200 (93.5%)	14 (6.5%)	*p* = 0.189
		66–75 years	129 (88.4%)	17 (11.6%)	
		76–85 years	59 (86.8%)	9 (13.2%)	
		≥85 years	7 (100%)	0 (0%)	
Gender		Male	207 (91.6%)	19 (8.4%)	*p* = 0.621
		Female	191 (90.1%)	21 (9.9%)	
Cancer		Lung	75 (75.8%)	24 (24.2%)	*p* < 0.0005
		Breast	46 (88.5%)	6 (11.5%)	
		Gastrointestinal	101 (98.1%)	2 (1.9%)	
		Liver	15 (100%)	0 (0%)	
		Gynecological	22 (91.7%)	2 (8.3%)	
		Head-Neck	14 (87.5%)	2 (12.5%)	
		Haematological	7 (100%)	0 (0%)	
		Prostate	17 (100%)	0 (0%)	
		Pancreas	47 (100%)	0 (0%)	
		Urological	25 (92.6%)	2(7.4%)	
		Other	25 (92.6%)	2 (7.4%)	
		Unknown	4 (100%)	0 (0%)	
Comorbidity	Cardiovascular	Yes	188 (88.7%)	24 (11.3%)	*p* = 0.166
		No	174 (93.0%)	13 (7.0%)	
	Bronco-pulmunary	Yes	33 (75.0%)	11 (58.0%)	*p* = 0.010
		No	329 (89.9%)	37 (10.1%)	
	Kidney disease	Yes	12 (85.7%)	2 (14.3%)	*p* = 0.379
		No	350 (90.9%)	35 (9.1%)	
	Liver disease	Yes	9 (100%)	0 (62.5%)	*p* = 1.0
		No	353 (90.5%)	37 (9.5%)	
	None		120/398 (30.2%)	6/40 (15.0)	*p* = 0.032
Treatment		Disease-oriented	264 (92.6%)	22 (7.4%)	*p* = 0.165
		Palliative Care	6 (22.2%)	21 (77.8%)	*p* = <0.0005

**Table 2:** Mean intensity (SD = standard deviation) of ESAS items.

	Mean	SD
Pain	3.83	3.094
Weakness	6.56	2.349
Nausea	1.86	2.754
Depression	3.78	3.283
Anxiety	4.33	3.152
Drowsiness	4.86	3.042
Dyspnea	4.71	3.126
Poor sleep	4.41	3.426
Poor appetite	4.41	3.646
Poor well-being	5.43	2.798
Total	43.87	16.996

**Table 3:** Differences in MDP items, ASS, HADS-A, HADS-D, and total HADS (see text) in patients with background dyspnea, background dyspnea and EB, and EB alone (see text).

	Background Dyspnea Only (13 pts)		Background and EB (27 pts)		EB Only (34 pts)		
	Mean	SD	Mean	SD	Mean	SD	
MDP effort	3.83	3.433	7.16	1.818	5.29	2.452	0.0003 A vs. B 0.002 C vs. B
MDP unpleasantness	4.50	2.876	7.69	1.463	5.84	2.541	<0.0005 A vs. B 0.001 C vs. B
MDP quality-intensity	18.17	14.534	27.40	8.124	21.53	12.371	0.014 A vs. B 0.038 C vs. B
MDP emotional	15.33	13.255	22.00	14.547	18.55	13.994	NS
BDI	32.58	15.635	42.70	11.671	34.19	17.610	0.027 A vs. B 0.035 C vs. B
ASS	9.17	5.006	9.81	3.962	8.59	5.200	NS
HADS-A	8.20	3.458	9.00	4.287	8.88	3.951	NS
HADS-D	8.20	2.658	8.91	3.146	10.12	4.512	NS
TOTAL HADS	16.40	5.680	17.91	6.886	19.00	6.468	NS
